# Noise-enhanced coding in phasic neuron spike trains

**DOI:** 10.1371/journal.pone.0176963

**Published:** 2017-05-04

**Authors:** Cheng Ly, Brent Doiron

**Affiliations:** 1 Department of Statistical Sciences and Operations Research, Virginia Commonwealth University, Richmond, VA 23284, United States of America; 2 Department of Mathematics, University of Pittsburgh, Pittsburgh, PA 15260, United States of America; 3 Center for the Neural Basis of Cognition, Pittsburgh, PA 15213, United States of America; Georgia State University, UNITED STATES

## Abstract

The stochastic nature of neuronal response has lead to conjectures about the impact of input fluctuations on the neural coding. For the most part, low pass membrane integration and spike threshold dynamics have been the primary features assumed in the transfer from synaptic input to output spiking. Phasic neurons are a common, but understudied, neuron class that are characterized by a subthreshold negative feedback that suppresses spike train responses to low frequency signals. Past work has shown that when a low frequency signal is accompanied by moderate intensity broadband noise, phasic neurons spike trains are well locked to the signal. We extend these results with a simple, reduced model of phasic activity that demonstrates that a non-Markovian spike train structure caused by the negative feedback produces a noise-enhanced coding. Further, this enhancement is sensitive to the timescales, as opposed to the intensity, of a driving signal. Reduced hazard function models show that noise-enhanced phasic codes are both novel and separate from classical stochastic resonance reported in non-phasic neurons. The general features of our theory suggest that noise-enhanced codes in excitable systems with subthreshold negative feedback are a particularly rich framework to study.

## Introduction

The neural transfer of synaptic inputs to output spike trains often involves cellular mechanisms with complex filtering properties. A majority of studies have focused on neurons with Class I excitability that show low frequency stimulus selectivity, or neurons with Class II excitability that have resonant properties [1–6]. However, recordings from neurons in the auditory brainstem [7–10], spinal cord [5, 11], and cerebellum [12] show a firing response preference for high frequency signals over low ones. Neurons with high pass selectivity are often labeled phasic, and are related to Class III excitability [4, 5, 13]. Phasic neural responses have received considerably less attention than Class I or Class II excitability and present a novel challenge in uncovering how fluctuations and signal combine to determine responses.

A common functional interpretation of phasic neuron responses is that they filter out low frequency inputs, which are presumably either distractors inputs, input noise, or are to be coded by another class of neurons. However, the responses from excitable systems are very nonlinear, and a high pass characterization obtained with a specific input structure may not generalize to other inputs. A classic example of this intuition is noise-enhanced coding, where moderate broadband fluctuations increase the information transfer of a weak signal [14–20]. Most models of excitable dynamics are one-dimensional, with artificial spike-reset mechanics erasing any memory of membrane dynamics prior to a spike [14–17]. For this reason these spike trains are guaranteed to have renewal statistics, greatly easing the analyses of noise-enhanced coding [15, 17]. One-dimensional neural dynamics, while nonlinear, nevertheless show base filter properties that are low-pass, in contrast to the high pass response of phasic neurons. In most models of phasic neurons, this high pass nature is achieved by a subthreshold negative feedback process, often from a low threshold K^+^ channel dynamic [5, 7, 10, 21–23]. Thus phasic neurons have dynamics that operate along a minimum of two dimensions, and noise induced spiking dynamics that have simple renewal statistics are not expected. Rather, the non-Markovian structure of stochastic responses in higher dimensional systems makes the analysis of noise-enhanced coding especially challenging [24–27]. Noise-enhanced coding in phasic neuron models have received recent attention [9, 10, 23, 28], however a sound theoretical framework that highlights the key differences between codes by phasic and non-phasic neurons has not been proposed.

We analyzed a simplified Fitzhugh-Nagumo neuron model with a state dependent timescale in the negative feedback recovery variable, capturing many of the core dynamic features of phasic neurons with a two-dimensional model. While the deterministic spike response of this model cannot code for low frequency signals (no matter the amplitude of the signal), the stochastic response shows a clear phase locking to these signals. We use a phenomenological model to expose the underlying mechanisms as a non-Markovian stochastic process that is distinct from classic stochastic resonance phenomena, yet generalizable to a broad class of excitable systems having subthreshold negative feedback.

## Results

### Fitzhugh-Nagumo phasic model

Consider the augmented Fitzhugh-Nagumo (FN) model (i.e., Class III excitability T [4]) receiving white noise input that mimics background activity:

$$\begin{array}{ccc} \tau_{v}\frac{dv}{dt} & = & v\left(0.1-v\right)\left(v-1\right)-w+I\left(t\right)+\sqrt{2D}\xi \left(t\right), \end{array}$$ (1)

$$\begin{array}{ccc} \frac{1}{\varepsilon (v)}\frac{dw}{dt} & = & v. \end{array}$$ (2)

**Fig 1:**
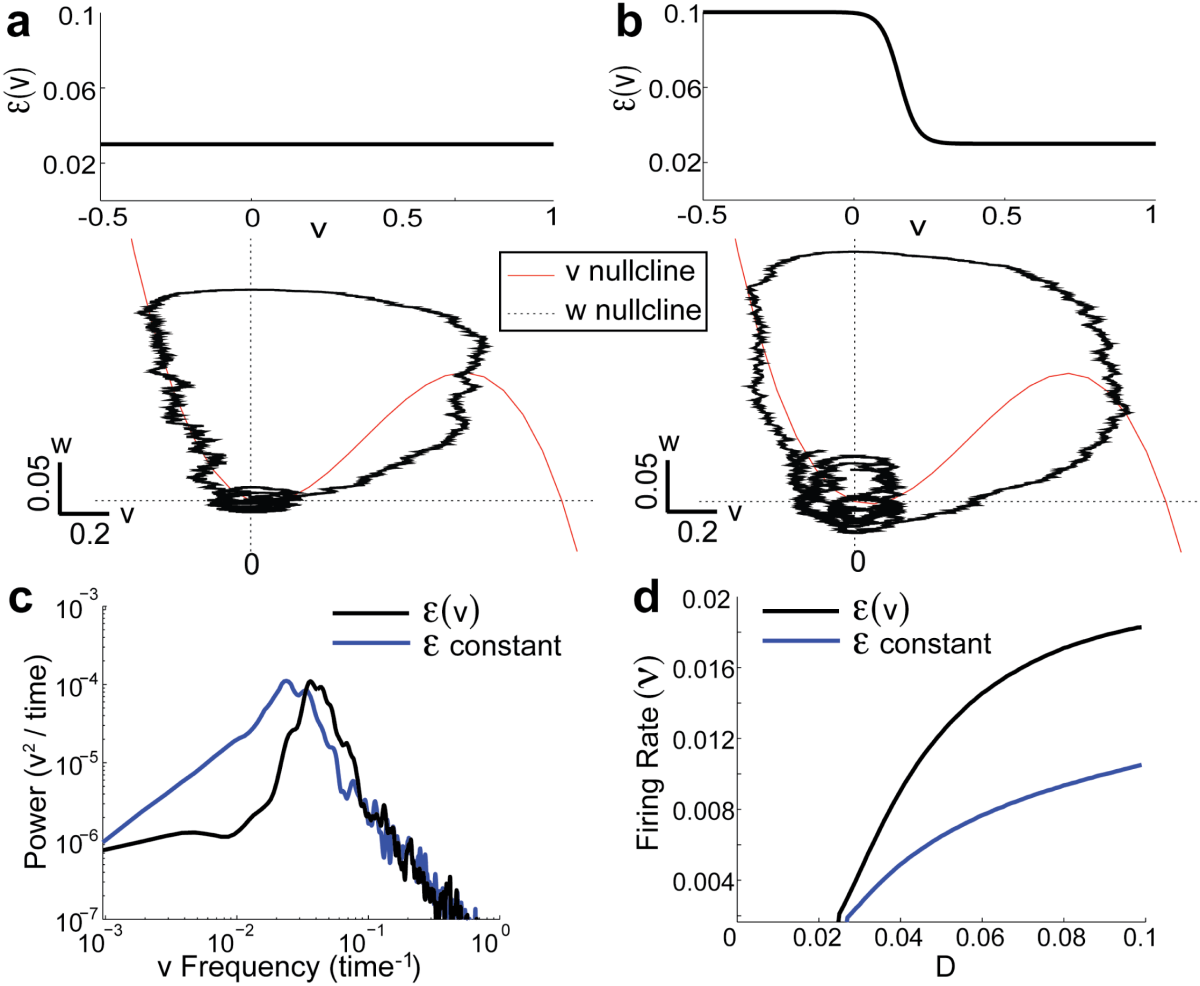
Phasic spiking with dynamic negative feedback. a) Typical trajectories in the phase plane for the *ε*-model (= 0.03, constant) and b) for $\varepsilon \left(v\right)=\frac{0.1+0.03e^{(v-0.15)/0.03}}{1+e^{(v-0.15)/0.03}}$. *D* = 0.03 in both (a) & b). c) Comparison of the power spectrum of *v*(*t*) with *D* = 0.03 for both curves. d) Comparison of the firing rates *ν* as a function of *D*. Throughout we define *ν* = 〈*y*(*t*)〉 where *y*(*t*) = ∑_*i*_ *δ*(*t* − *t*_*i*_) and *t*_*i*_ is the time of the *i*^*th*^ trajectory crossing of *w* = *w*_*c*_ from below (*w*_*c*_ = 0.14 for *ε*(*v*)-model and *w*_*c*_ = 0.15 for the *ε*-model).

Here *v* models the membrane potential of a neuron, and *w* models a refractory variable responsible for action potential repolarization. In this model the *w*-nullcline is vertical (Fig 1a, bottom) so that the rest state *v* = 0 is always stable for all *I*(*t*) = *I* constant. This is in contrast to the standard Fitzhugh-Nagumo model, where a slanted *w*-nullcline causes the rest state to loose stability via a subcritical Hopf bifurcation when *I* is increased. We are interested in the response of the model to time varying input, where *I*(*t*) is a dynamic signal and *ξ*(*t*) is a Gaussian noise source of intensity *D* obeying 〈*ξ*(*t*)〉 = 0 and 〈*ξ*(*t*)*ξ*(*t*′)〉 = *δ*(*t* − *t*′). The variables *τ*_*v*_ and *ε*^−1^ represent the timescales of *v* and *w*, respectively.

Before we consider the response to dynamic input we note that real phasic neurons have a strong subthreshold negative feedback (often a voltage gated K^+^ channel), whose timescale determines the high pass property of the spike train response [7–10]. In most models this is captured by an auxiliary variable [9, 10, 21] that often adds more dimensions to the underlying model. However, in our model we introduce dynamics to the timescale of the negative feedback *w* by letting *ε*^−1^ depend on *v*. We refer to our dynamic timescale model as the *ε*(*v*)-model, as opposed to the constant timescale *ε*-model. Note that Lundstrom et al. [28] also introduced a voltage dependent time-scale *ε*(*v*) in a 2D system based on a reduced Hodgkin-Huxley model developed by Rush & Rinzel [29] (see Discussion). In general we take *τ*_*v*_ ≪ *ϵ*(*v*)^−1^ for all *v* so that action potential dynamics can occur; however, the *ε*(*v*)-model has a faster *w* timescale (larger *ϵ*(*v*)) when the model is not engaged in a spike (*cf.* Fig 1a and 1b, top). This accentuates circular paths in the (*v*, *w*) phase-plane near rest (*v*, *w*) = (0, 0) (*cf*. Fig 1a and 1b; bottom). This circular flow is reminiscent of mixed-mode oscillations, a widespread phenomena in many scientific disciplines [30–33] characterized by distinct oscillations of various amplitudes. The subthreshold oscillatory dynamic gives an overall increased rhythmic dynamic to the *ε*(*v*)-model, as compared to the constant *ε*-model (Fig 1c). However, an important distinction between the two models is that the *ε*(*v*)-model’s spike train firing rate *ν* has a higher sensitivity to changes in the noise intensity *D* (Fig 1d). Taken together, many of the known features of phasic neurons are captured by a two-dimensional FN model with a modification of the time-scale of the recovery variable (*ε*(*v*)-model).

**Fig 2:**
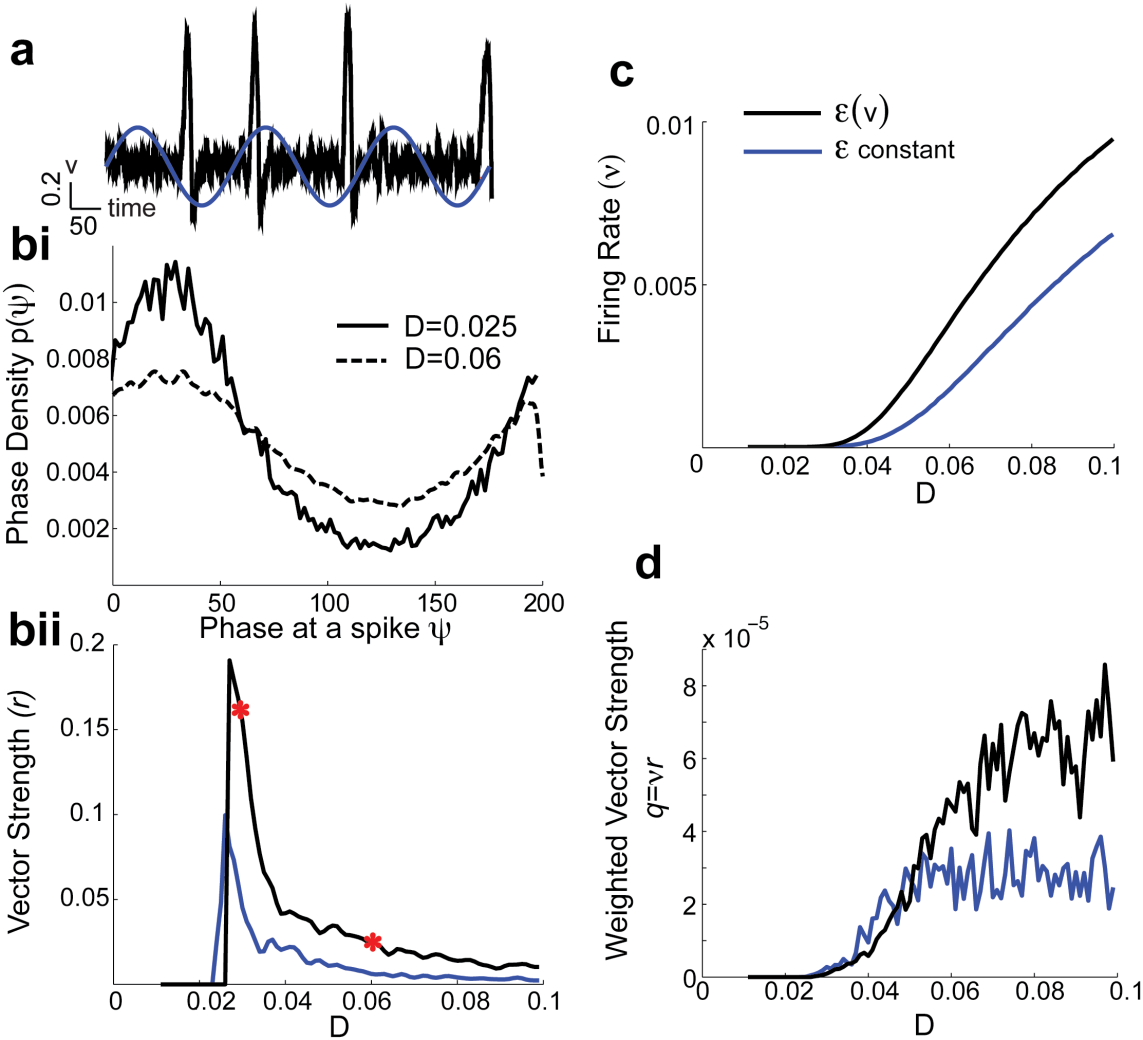
Phasic Fitzhugh-Nagumo model responses lock to slow sinusoidal input. a) Model responses to low frequency sinusoidal signals (*φ* = 0.005 with *A* = 0.01). *v*(*t*) superimposed with *I*(*t*) = *A* sin(2*πφt*) for *ε*(*v*)-model. bi) The phase density (i.e., phase of sinusoidal input at a spike), *p*(*ψ*), in the *ε*(*v*)-model with two different noise levels (corresponding to red asterisks in bii)). bii) The vector strength, *r*, (see Eq (3)) as a function of noise level *D*. The *ε*(*v*) model response is better locked to sinusoidal input compared to that from the *ε*-constant model. The strength of the noise has to be large enough to ellicit a response with slow sinusoidal input. c) Firing rates are higher with *ε*(*v*) modification and more sensitive to noise than when *ε* is constant. (d) The *q*-values are larger for a wider range of noise values *D* in the *ε*(*v*)-model because the firing rate higher (c) and it is better locked to the input.

We next compare the spike train coding of low frequency sinusoidal inputs *I*(*t*) = *A* sin(2*πφt*) (*φ* ≪ 1) for both the *ε*(*v*) and *ε*-models (Fig 2a). When *D* = 0 and *φ* is sufficiently small both models do not produce a spike, even for very large *A* (Fig 2c). However, for signals in the presence of noise (*D* > 0) both phasic models fire and the increased sensitivity to *D* of the *ε*(*v*)-model persists when *A* > 0. To measure the tendency for spike responses to track dynamic inputs we define the vector strength *r* ∈ [0, 1] as:

$$\begin{array}{c} r={\left({\left|\int_{0}^{T}p\left(\psi \right)\cos\left(2\pi \varphi \psi \right)\;d\psi \right|}^{2}+{\left|\int_{0}^{T}p\left(\psi \right)\sin\left(2\pi \varphi \psi \right)\;d\psi \right|}^{2}\right)}^{1/2}. \end{array}$$ (3)

Here *p*(*ψ*) is the phase density (probability density of the random phase of the sinusoid exactly at the time of a spike) and *T* = 1/*φ*. Vector strength is a common measure of the degree of phase-locking to a periodic signal [34–37]. Higher values of *r* indicate better phase locking than lower values, and the *ε*(*v*)-model shows a much larger *r* over a range of *D* than the *ε*-model (see Fig 2b). A signal is well encoded if the spike responses are both phase-locked (high *r*) and dense in time (high firing rate *ν*), and we thus consider the vector strength *weighted* by firing rate *ν*:

$$\begin{array}{c} q≔\nu r. \end{array}$$ (4)

The large firing rate and phase locking in the *ε*(*v*)-model combine to dramatically increase the overall *q* for a range of *D* values compared to the *ε*-model (Fig 2d). Thus, the inclusion of a *v*-dependent timescale of the negative feedback increases the sensitivity of our phasic model to noisy fluctuations. For moderate *D* the enhanced sensitivity does not impede, yet it rather enhances, the encoding of a slow signal by the spike train response.

**Fig 3:**
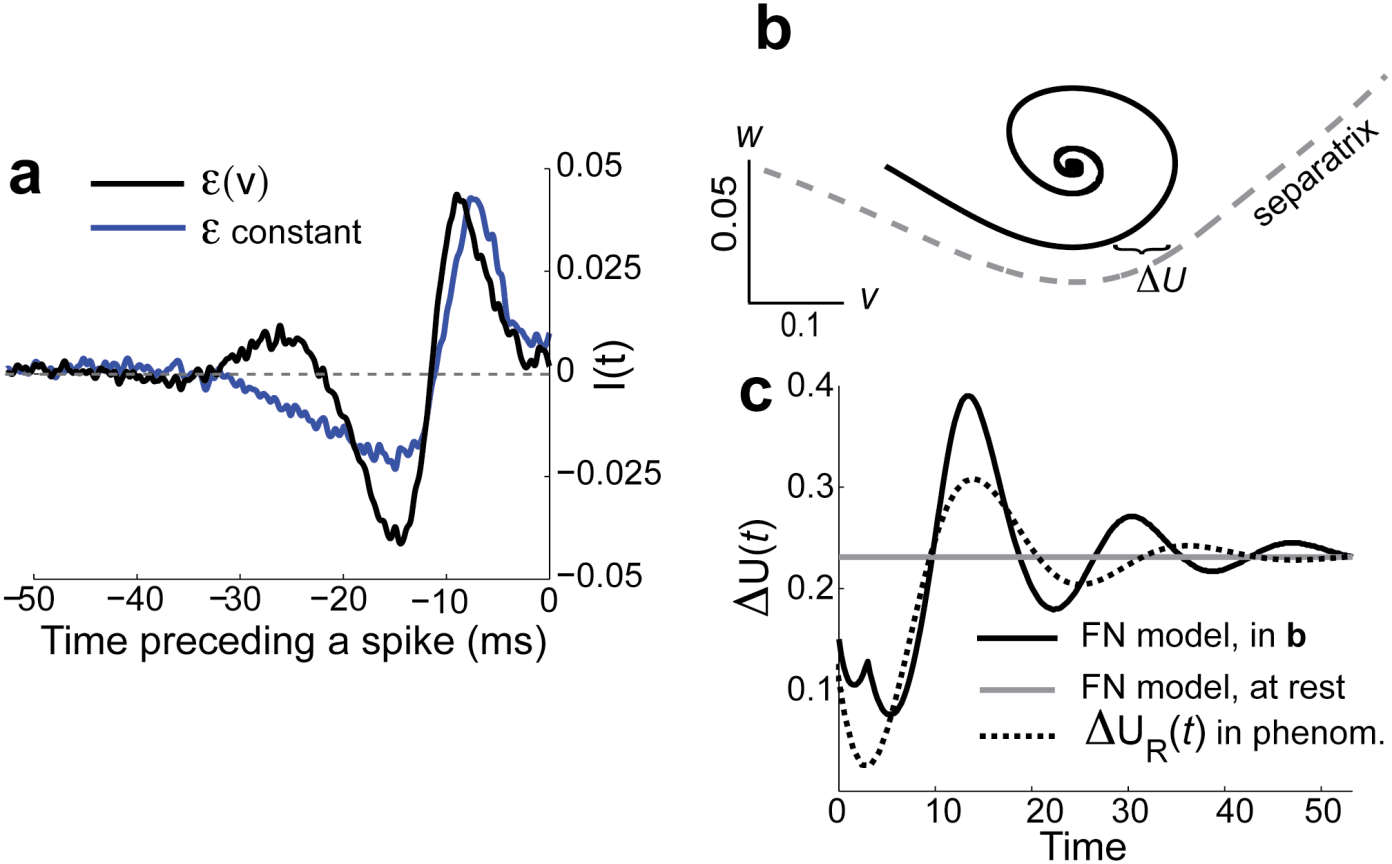
Low dimensional FN model motivates reduced phenomenological model. a) 〈*ξ*(*t*)|*t*_*s*_ = 0〉 with *I*(*t*) = 0 for *ν* = 0.0045 (*D* = 0.03 for *ε*(*v*)-model and *D* = 0.069 for *ε*-model), scaled to have the same maximum [8]. This demonstrates that the neuron prefers input that is hyperpolarizing, quickly followed by depolarizing input. b) The trajectory in the (*v*, *w*) phase plane in gray, starting at (*v*, *w*) = (−0.2, 0) with *I*(*t*) = *D* = 0. The minimum distance to the separatrix (gray-dashed) in the *v* direction is Δ*U*. c) The evolution of Δ*U* in (b) (solid black), and initial Δ*U* (gray) are plotted, showing the benefit of hyperpolarizing input. Scaled Δ*U*_*R*_(*t*) that is used in reduced model (dotted black) is shown for comparison.

The sensitivity of *q* to the *v* dependence of *ε* shows that subtle changes in subthreshold integration can have a large impact of spike train coding. To measure this we compute 〈*ξ*(*t*)|*t*_*spike*_ = 0〉 for *t* < 0, often termed the spike triggered average [38]. The spike response of the *ε*(*v*) model shows a preference for a strong hyperpolarizaing (negative) input that quickly depolarizes near the spike time (Fig 3a, black curve), in agreement with *in vitro* experiments of phasic neurons [7, 8] (also see Fig 6 in [11] for recent experimental verification of this phenomena). While the *ε* model shows a similar selectivity (Fig 3a, blue curve), the effect is diluted compared to that shown by the *ε*(*v*) model.

To understand the how *ε*(*v*) shapes the spike triggered average we first considered the relaxation dynamics of the deterministic trajectory (*v*(*t*), *w*(*t*)) from an initial hyperpolarized *v* (Fig 3b). The trajectory shows that the distance to the separatrix in the *v* (horizontal) direction is often smaller than when at rest (Fig 3c, black curve). The increased rotational flow near the rest state for the *ε*(*v*)-model transiently lowers the effective ‘threshold’ for spike initiation, an attribute well-known in several neural models [4, 28]. This gives an intuitive understanding of why the combination of hyperpolarization followed by depolarization evokes spike responses. This selectivity of spike dynamics has yet to be incorporated into a theory of noise-enhanced coding. Note that these dynamics are only somewhat related to the post-inhibitory rebound or anode break excitation whereby a spike can be initiated with a quick removal of inhibition [29]. Here, the inhibition is transient as opposed to post inhibitory rebound that initially requires sustained inhibition.

We next propose a reduced framework that builds on past models of stochastic resonance [14–19] and exposes how noise enhances coding to a larger degree in phasic than non-phasic neurons.

### Phenomenological model of phasic neuron coding

Consider a phenomenological model of the spiking activity, where the *v* dynamics are captured by a one-dimensional potential well *U*(*v*, *t*):

$$\begin{array}{c} \frac{dv}{dt}=-U^{\prime }\left(v,t\right)+\sqrt{2D}\xi \left(t\right), \end{array}$$ (5)

where ′ denotes differentiation with respect to *v*. For exposition purposes, we ignore the external sinusoidal signal (for the inclusion of a sinusoidal signal, see Materials and Methods section). In what follows we take *v* = 0 as a stable state and neglect the local dynamics about *v* = 0. We consider only large, infrequent fluctuations that force *v* to exceed one of two threshold values: *v*_*R*_ > 0 (right of *v* = 0) and *v*_*L*_ < 0 (left of *v* = 0). When *v* crosses *v*_*R*_ a spike occurs and *v* is reset to 0. The energy required for this to occur is Δ*U*_*R*_ = *U*(*v*_*R*_) − *U*(0), we refer to Δ*U*_*R*_ as the right barrier. The second threshold *v*_*L*_, with energy barrier Δ*U*_*L*_ = *U*(*v*_*L*_) − *U*(0), models the activation of the subthreshold negative feedback that is recruited by a hyperpolarizing fluctuation in the *ε*(*v*) FN model (see Fig 3b). We incorporate this feedback as a transient dynamic to the spike threshold. Specifically, if *v* crosses *v*_*L*_ at time *t* = 0 then the right energy barrier has a damped oscillatory dynamic Δ*U*_*R*_(*t*) for *t* > 0 to capture the distance to threshold in the *ε*(*v*) FN model (Fig 3c, solid black curve). In the phenomenological model, we let Δ*U*_*R*_(*t*) = *v*_*R*_ − 1.4sin(0.8*π*(*t* + 0.15))/*e*^0.8(*t*+0.25)^ (Fig 3c, dotted curve) to qualitatively capture the distance to the separatrix in the *ε*(*v*) FN model (compare the curves in Fig 3c). In effect, a sufficiently large hyperpolarization will impact the likelihood of a future depolarization to cause a spike threshold crossing. This effect is reminiscent of mixed-mode oscillations where small subthreshold oscillations facilitate large suprathreshold oscillations [30–33]. From these assumptions we can build a theory for spike dynamics based on ideas from renewal theory [39, 40], an approach has been successfully used to study a variety of systems [41], including non-phasic neural models [18, 19, 28, 42–44].

Let *T*_*L*_ ∈ [0, ∞) denote the random time that *v* crosses the left barrier *v*_*L*_, ignoring crossings of *v*_*R*_ for the moment. In the model without a time-varying input current, the rate of crossing the left barrier, termed the hazard function *H*_*L*_, is constant in time. We assume that *H*_*L*_ necessarily increases with noise level *D*, and decreases with barrier height Δ*U*_*L*_. In many models a decent approximation is given by the Arrhenius law for small noise *D* ≪ 1: *H*_*L*_ ≈ *γe*^−Δ*U*_*L*_/*D*^ [28, 41, 42, 44]. Assuming this renewal framework for left barrier crossing we may compute other related quantities of interest, namely the survivor function *S*_*L*_(*t*) and the probability distribution of the inter-crossing intervals *f*_*L*_(*t*). For small *dt* we have the following relations:

$$\begin{array}{ccc} H_{L} & ≔ & \mathrm{Pr}\left(t\leq T_{L}\leq t+dt\right)/dt=H\left(\Delta U_{L},D\right),\\ S_{L}\left(t\right) & ≔ & \mathrm{Pr}\left(T_{L}>t\right)=e^{-H_{L}t},\\ f_{L}\left(t\right) & ≔ & \mathrm{Pr}\left(t\leq T_{L}\leq t+dt|T_{L}\notin \left(0,t\right)\right)/dt=H_{L}e^{-H_{L}t}. \end{array}$$ (6)

Ignoring the left barrier for now, consider the times *T*_*R*_ ∈ [0, ∞) that *v* crosses the moving right barrier. If Δ*U*_*R*_(*t*) varies slowly (i.e., in the *ε*(*v*) FN model when subthreshold), the time-varying hazard function *H*_*R*_(*t*) is well-approximated by an inhomogenous Poisson process. We assume further that the only temporal dynamics of *H*_*R*_ arise via the moving barrier Δ*U*_*R*_(*t*). The associated hazard, survivor, and inter-crossing interval distribution are given by

$$\begin{array}{ccc} H_{R}\left(t\right) & ≔ & \mathrm{Pr}\left(t\leq T_{R}\leq t+dt\right)/dt=H\left(\Delta U_{R}\left(t\right),D\right),\\ S_{R}\left(t\right) & ≔ & \mathrm{Pr}\left(T_{R}>t\right)=e^{-\int_{0}^{t}H_{R}\left(s\right)\;ds},\\ f_{R}\left(t\right) & ≔ & \mathrm{Pr}\left(t\leq T_{R}\leq t+dt|T_{R}\notin \left(0,t\right)\right)/dt=H_{R}\left(t\right)e^{-\int_{0}^{t}H_{R}\left(s\right)\;ds}. \end{array}$$ (7)

To more accurately capture the sensitivity of the full FN models to *D* we set *H*(Δ*U*, *D*): = 5*e*^−3Δ*U*^1.5^/*D*^, with Δ*U*_*L*_ = 0.9 and *v*_*R*_ = 1.5. Our theory is not sensitive to this choice, and the subsequent derivations in the **Materials and Methods** section are with a general *H*(Δ*U*, *D*).

Combining both left and right barriers, the crossing statistics are non-Markovian, presenting analytical difficulties that are addressed in the **Materials and Methods** section. We analytically obtain an equation for the probability density function of time between spikes, *T*: *f*(*t*)*dt* = Pr(*t* ≤ *T* ≤ *t* + *dt*|*T* ∉ (0, *t*)) (see Eq (24)), yielding the firing rate:

$$\begin{array}{c} \nu ={\left(\int_{0}^{\infty }tf\left(t\right)\;dt\right)}^{-1}. \end{array}$$ (8)

The equation for the phase density *p*(*ψ*) (Eq (23)) and subsequently the vector strength *r* (Eq (25)) are also derived in the **Materials and Methods** section.

**Fig 4:**
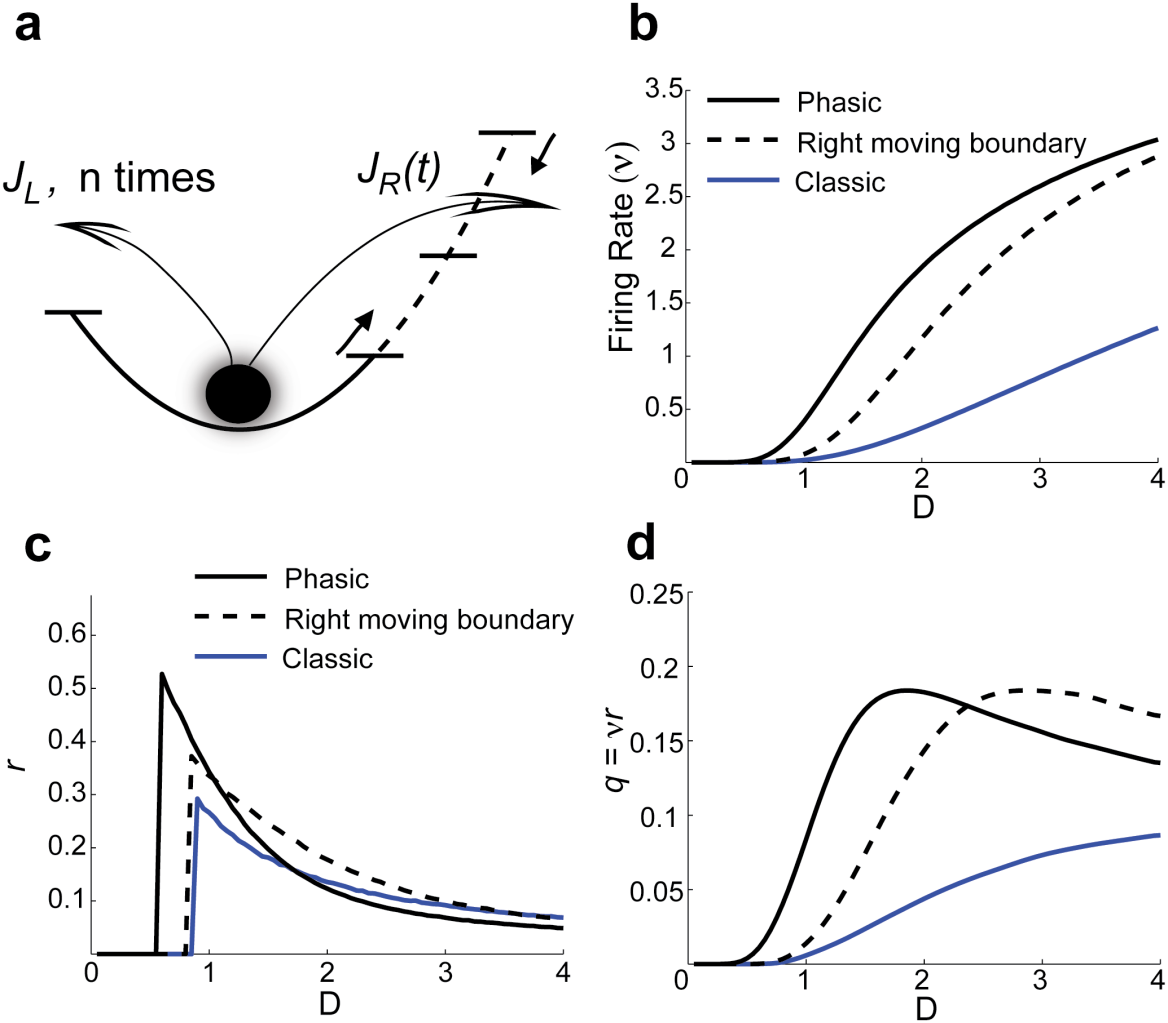
Phenomenological model dissects core mechanisms of noise-enhanced coding. a) In the reduced system, leftward exits reset the right barrier, effectively lowering it; the right barrier evolves according to the dotted curve Δ*U*(*t*) in Fig 3c. b) The firing rate dependence on *D* for the non-phasic model (classic, with constant right boundary and no left boundary), a model with a dynamic spike threshold only, and the full phasic model (see Eqs (8) and (24)). c) The vector strength *r* computed by the analytic formulas for all 3 models (Eq (25)). d) The noise-enhanced encoding *q* of a sinusoidal input for the three models. Throughout we set $H\left(\Delta U_{R},D\right)≔5e^{-3\Delta U_{R}^{1.5}/D}$ to capture the sensitivity of *ν* to noise (cf. Figs 1d) and 4b), and *φ* = 0.1.

This phenomenological model enables the parsing of how the components (negative feedback, moving threshold, etc.) can lead to enhanced encoding of slow signals. We shed light on these dynamics by comparing three different models (Fig 4):

1. The **Phasic** model we have just described where left barrier crossing resets the moving height Δ*U*_*R*_(*t*).
2. Dynamic right barrier Δ*U*_*R*_(*t*) after a spike reset, yet without a left barrier to reset Δ*U*_*R*_(*t*) (i.e, *J*_*L*_ → 0 so only the first term appears in Eq (11)). We refer to this model as the **Right moving boundary** model.
3. A non-phasic model with no left barrier, and Δ*U*_*R*_(*t*) height only changes with sinusoidal input, as previously studied [14–19]. We refer to this last model as the **Classic** model.

Over a large range of *D*, the firing rate *ν* and noise sensitivity *dν*/*dD* of the phasic model (1) are larger than the other two models (Fig 4b; here *A* ≪ 1 so the rates are the same with and without sinusoidal input). Thus, lowering of Δ*U*_*R*_(*t*) by hyperpolarization promotes spike discharge and increases firing rate sensitivity to noise, as observed in the FN model (Fig 2c). More importantly, the reduced phasic model (1) encodes low frequency sinusoidal signals with higher *q* values than the other models (Fig 4d), also observed in the FN model (Fig 2d). We remark that for small *D*, model (2) is very different from the phasic model (1) since *ν* is low and multiple left barrier resets between spikes are likely, however for large *D* the model responses approach one another. In total, our reduced framework shows how the subthreshold negative feedback in phasic systems promotes and enhances coding of slow signals with background noise.

## Discussion

In deterministic frameworks phasic neurons are high pass systems, with a lack of response to sufficiently low frequency inputs of even large amplitude. However, we have shown that in the presence of noise, the coding of phasic systems is rich, allowing phasic systems to easily code for low frequency inputs. Although others have demonstrated computationally that background fluctuations can lead to better coding of slow signals [9, 10, 28], we capture the essence of this effect with a 2D planar model which subsequently motivates a reduced phenomenological description of the dynamics. Our analysis specifically enables parsing of the various pieces (i.e., removal of negative feedback, threshold dynamics) that contribute to the observed effect. Despite its simplicity, our reduced point process description shows how the subthreshold negative feedback inherent in phasic neuron activity is an important feature that enhances the overall capacity for noise to enhance the coding of slow signals. Our theoretical analysis shows how noise-enhanced coding in phasic systems is mechanistically separate from classic stochastic resonance [14–19], and is a novel form of noise-enhanced coding.

Introducing a voltage-dependent time constant for the inactivation variable has been beneficial in explaining other electrophysiological recordings. Lundstrom et al. [28] used an energy barrier model to understand how noise can cause more firing in Class III neurons and other kinds of neurons in a variety of cortical areas. Specifically, they explained how *ε*(*v*) slows the subthreshold voltage dynamics that can lead to increase in firing rate with noise. They primarily focused on non-phasic neurons with the analysis focused on type A (insensitivity to noise with large mean current) and type B+ (sensitivity to noise but repetitive firing as mean current increases). As such they did not dissect the role of noise as specifically as we did, whereby negative fluctuations remove the slow negative feedback and primes the neuron to spiking (see the spike-triggered average of the *ε*(*v*)-phasic neuron). Recently, Ratte et al. [11] provided detailed analyses of various response dynamics in different types of neurons using phase plane analyses in models and with *in vitro* experiments. They also found similar behaving phasic-type neurons (‘differentiators’) in the spinal cord of rodents that required hyperpolarization following by depolarization to initiate spikes.

How fluctuations drive subthreshold nonlinear dynamics is a potentially fruitful avenue for mixed mode oscillator systems. However, this has been understudied because the intrinsic dynamics are often complicated. Work by Gai, Rinzel and colleagues [9, 10, 23] demonstrated this similar effect of noise enhancing the ability of class III neurons to encode low frequency signals. They used a combination of in vitro experiments and computational modeling (4D Hodgkin-Huxley model), but did not precisely dissect the underlying details of how noise removes the negative feedback (i.e., a fast Potassium current) to promote spiking the way we have in two-dimensional model that inspired our phenomenological model. Our work shows how even in a simple framework, the impact of noise on coding in these rich systems can be dramatic.

Although we have only considered a single sinusoidal amplitude and frequency for exposition purposes, our results will hold with other parameter values as long as these two parameters are relatively small. Examination of the various entities in this paper with other parameter values yield the same story (also see Supporting information, Fig A in S1 Text, Fig B in S1 Text, and Fig C in S1 Text). Determining the exact critical point where our results no longer hold (e.g., increasing the frequency and/or amplitude) is difficult because the analysis of the phenomenological model relies on these assumptions. Such investigations are beyond the scope of this study but may yield interesting results.

An overall better understanding of the dynamics of how phasic neurons encode signals is important in the context of coincidence detection [23, 45] known to be important for example in the auditory system; in fact, some refer to phasic neurons as ‘coincidence-detector’ neurons [45].

Our work highlights the impact of negative feedback in the subthreshold membrane dynamics on spike discharge. Our phenomenological model shows a non-Markovian character of the membrane dynamics, however this memory is not carried over after spike discharge. As a result we would expect that inter-spike interval sequence to be renewal (i.e successive intervals are independent of one another). This should be contrasted with neuronal models with spike driven slow adaptation [46]. The time scale of the negative feedback produces negative serial inter-spike interval correlations, making the spike train process formally non-renewal [47]. This non-renewal character also increases information transfer about static and dynamic signals in these systems (Chacron 2001, 2004). Phenomenological models of these spike dynamics involve time dependent spike thresholds, which are similar to the time dependent left barrier in our model [25, 47]. A topic of further study would be to combine these two models so that sub-threshold and supra-threshold negative feedback interact.

## Materials and methods

The next three sections contain the derivation of the probability density of time between spikes *f*(*t*)*dt* = Pr(*t* ≤ *T* ≤ *t* + *dt*|*T* ∉ (0, *t*)) in the reduced phenomenological model in the following order for exposition purposes:

- Without sinusoidal input (Eq (12))
- With slow sinusoidal input that resets after a spike (Eq (19)); the sinusoidal input starts with the same phase, i.e., endogenous input
- Finally, with slow sinusoidal input that does not reset after a spike (Eq (24)), i.e., exogenous input

Also, the equation for the phase density *p*(*ψ*) and thus the vector strength *r* are derived for the reduced phenomenological model (see in Eqs (23) and (25), respectively).

### Phenomenological model without external input

We are interested in the spiking statistics of the phenomenological model with both a left and right barrier (see Fig 4a). Recall the definitions of the associated hazard, survivor, and density of event times in Eqs (6) and (7). Without loss of generality assume that a spike event has happened at time *t* = 0. Let *T* ∈ [0, ∞) be the random time of the neuron’s next spike. We are interested in the calculating the inter-spike interval density for *T*; *f*(*t*) = Pr(*t* ≤ *T* ≤ *t* + *dt*|*T* ∉ (0, *t*))/*dt*. The probability of *T*_*R*_ (crossing the **R**ight boundary first after spike reset) and *T*_*L*_ (crossing the **L**eft boundary first after spike reset) simultaneously occurring goes to 0 as *dt* → 0, this gives that the probability per unit time of crossing the right barrier (firing) without first crossing the left barrier is equal to: *S*_*L*_(*t*)*f*_*R*_(*t*). That is, the membrane potential dynamics must survive the left barrier up to time *t* (with probability *S*_*L*_(*t*)), then cross the right barrier (with probability density *f*_*R*_(*t*)). We remark that these are independent events in this formulation because we ignore small amplitude fluctuations, and only consider large fluctuations that cause barrier crossings. Similarly, the probability of crossing the left barrier without ever crossing the right threshold is: *S*_*R*_(*t*)*f*_*L*_(*t*). For convenience, label these quantities *J*_*R*_(*t*) and *J*_*L*_(*t*), respectively.

$$\begin{array}{ccc} J_{R}\left(t\right) & ≔ & S_{L}\left(t\right)f_{R}\left(t\right)=H_{R}\left(t\right)\exp\left(-\int_{0}^{t}H_{R}\left(s\right)\;ds-tH_{L}\right) \end{array}$$ (9)

$$\begin{array}{ccc} J_{L}\left(t\right) & ≔ & S_{R}\left(t\right)f_{L}\left(t\right)=H_{L}\exp\left(-\int_{0}^{t}H_{R}\left(s\right)\;ds-tH_{L}\right) \end{array}$$ (10)

As stated earlier, the neuron can fire without ever crossing the left barrier, which has probability (per unit time): *J*_*R*_(*t*). However, it can also fire by first crossing the left barrier at time *t*′ < *t* exactly once, and then crossing the right barrier at time *t*. The probability (per unit time) of this occurring is:

$$\begin{array}{c} \int_{0}^{t}J_{L}\left(t-t^{\prime }\right)J_{R}\left(t^{\prime }\right)\;dt^{\prime } \end{array}$$

because the times of these events are independent random variables. Continuing in this fashion, we see that the neuron can fire by crossing the left barrier *n* (*n* = 0, 1, 2, …) times before it crosses the right barrier, which has probability (per unit time) equal to an *n*-fold convolution:

$$\begin{array}{c} \int_{0}^{t}J_{L}\left(t-t^{\prime }\right)\int_{0}^{t^{\prime }}J_{L}\left(t^{\prime }-t^{\prime \prime }\right)\cdots \int_{0}^{t^{\left(n-1\right)}}J_{L}\left(t^{\left(n-1\right)}-t^{\left(n\right)}\right)J_{R}\left(t^{\left(n\right)}\right)\;dt^{\left(n\right)}\;dt^{\left(n-1\right)}\cdots \;dt^{\prime }. \end{array}$$

Summing over *n* = 0, 1, 2, ‥ gives all the different ways the neuron can spike at *t* (schematically shown in Fig 4a), yielding:

$$\begin{array}{ccc} f(t) & = & J_{R}\left(t\right)+\int_{0}^{t}J_{L}\left(t-t^{\prime }\right)J_{R}\left(t^{\prime }\right)\;dt^{\prime }\\ & & +\int_{0}^{t}J_{L}\left(t-t^{\prime }\right)\int_{0}^{t^{\prime }}J_{L}\left(t^{\prime }-t^{\prime \prime }\right)J_{R}\left(t^{\prime \prime }\right)\;dt^{\prime \prime }\;\;dt^{\prime }+\cdots \\ & = & J_{R}\left(t\right)+\int_{0}^{t}J_{L}\left(t-t^{\prime }\right)\left[J_{R}\left(t^{\prime }\right)+\int_{0}^{t^{\prime }}J_{L}\left(t^{\prime }-t^{\prime \prime }\right)J_{R}\left(t^{\prime \prime }\right)\;dt^{\prime \prime }+\cdots \right]\;dt^{\prime } \end{array}$$ (11)

Notice the terms in the square brackets above are exactly *f*(*t*′):

$$\begin{array}{c} f\left(t\right)=J_{R}\left(t\right)+\int_{0}^{t}J_{L}\left(t-t^{\prime }\right)f\left(t^{\prime }\right)\;dt^{\prime } \end{array}$$ (12)

Taking the Fourier Transform and applying the convolution theorem to this equation, we see that the characteristic function $\hat{f}\left(\omega \right)≔\int_{0}^{\infty }e^{-i\omega t}f\left(t\right)\;dt$ is:

$$\begin{array}{c} \hat{f}\left(\omega \right)=\frac{{\hat{J}}_{R}\left(\omega \right)}{1-{\hat{J}}_{L}\left(\omega \right)} \end{array}$$ (13)

from which we can obtain the ISI density assuming $\hat{f}$ is uniquely invertible

$$\begin{array}{c} f\left(t\right)=\frac{1}{2\pi }\int_{-\infty }^{\infty }e^{i\omega t}\hat{f}\left(\omega \right)\;d\omega \end{array}$$ (14)

A similar derivation for the transition probabilities in a two-state non-Markovian system has been proposed ([26]; Eqs (14)–(32)); however we have intertwined the crossing of one barrier to the dynamics of a second.

### Phenomenological model with sinusoidal input

Assume the system (Eq (5)) now has a weak sinusoidal (*A* ≪ *v*_*R*_) input with slow frequency *φ* ≪ 1:

$$\begin{array}{c} \frac{dv}{dt}=-\Delta U^{\prime }\left(v,t\right)+A\sin\left(2\pi \varphi t\right)+\sqrt{2D}\xi \left(t\right) \end{array}$$ (15)

With slow frequencies, the dynamics are well-approximated by the quasi-static limit (i.e., that *v*(*t*) is its steady-state value that slowly changes in time). Again, we consider the reduced phenomenological model discussed in the last section. The quasi-static approximation is thus equivalent to modulating the left and right barriers sinusoidally with an amplitude *A*. Here the left barrier changes in time: Δ*U*_*L*_ − *A* sin(2*πφt*), and the right barrier is Δ*U*_*R*_(*t*) − *A* sin(2*πφt*).

The formulas developed in Eqs (6)–(14) for the ISI density *f*(*t*) can be generalized for Eq (15), except now both left and right hazard functions vary in time

$$\begin{array}{ccc} H_{L}\left(t\right) & = & H(\Delta U_{L}-A\sin\left(2\pi \varphi t\right),D)\\ H_{R}\left(t\right) & = & H(\Delta U_{R}\left(t\right)-A\sin\left(2\pi \varphi t\right),D). \end{array}$$ (16)

The probability (per unit time) of exiting the left and right barrier are now:

$$\begin{array}{ccc} J_{L}\left(t\right) & = & S_{R}\left(t\right)f_{L}\left(t\right)=H_{L}\left(t\right)\exp\left(-\int_{0}^{t}H_{R}\left(s\right)+H_{L}\left(s\right)\;ds\right)\\ J_{R}\left(t\right) & = & S_{L}\left(t\right)f_{R}\left(t\right)=H_{R}\left(t\right)\exp\left(-\int_{0}^{t}H_{R}\left(s\right)+H_{L}\left(s\right)\;ds\right) \end{array}$$ (17)

The Fourier transform of the ISI density is (recall Eqs (11) and (12):

$$\begin{array}{c} \hat{f}\left(\omega \right)=\frac{{\hat{J}}_{R}\left(\omega \right)}{1-{\hat{J}}_{L}\left(\omega \right)}. \end{array}$$ (18)

from which we can obtain the ISI density if the system is renewal (i.e., here we assume *I*(*t*) is reset to *A* sin(2*πφt*) after every spike)

$$\begin{array}{c} f\left(t\right)=\frac{1}{2\pi }\int_{-\infty }^{\infty }e^{i\omega t}\hat{f}\left(\omega \right)\;d\omega . \end{array}$$ (19)

### Theory for the phase density and vector strength

The last section showed the derivation of the ISI density in the quasi-static approximation assuming the signal is endogenous (i.e., always start with the same *I*(*t*) = *A* sin(2*πφt*) after a spike). However, physiological signals are exogenous so that after spike reset there are different initial phases *ψ*_0_ ∈ [0, *T*) of where the signal starts: *I*(*t*) = *A* sin(2*πφ*(*t* + *ψ*_0_)) (here $T=\frac{1}{\varphi }$), adding further complications for analytic characterization. The subsequent theory for the probability density of the phase at a spike: *p*(*ψ*); $\int_{0}^{T}p\left(\psi \right)\;d\psi =1$, i.e., the cycle histogram, that we use is based on the work of Shimokawa et al. [48].

We can calculate a family of ISI densities conditioned on a starting phase *ψ*_0_: *f*(*t*|*ψ*_0_) with *I*(*t*) = *A* sin(2*πφ*(*t* + *ψ*_0_)). The probability density function of firing at phase *ψ* given that the starting phase is *ψ*_0_ is (see Eq (19))

$$\begin{array}{c} g\left(\psi |\psi_{0}\right)=\frac{1}{2\pi \varphi }\sum_{k=0}^{\infty }f\left(kT+\psi -\psi_{0}|\psi_{0}\right); \end{array}$$ (20)

it also satisfies: $\int_{0}^{T}g\left(\psi |\psi_{0}\right)\;d\psi =1$ for all *ψ*_0_. Let us define an operator L that maps the probability density of initial phases after the (*n* − 1)^th^ spike *p*_*n*−1_(*ψ*_0_) to a probability density of phases at the *n*^th^ spike *p*_*n*_(*ψ*):

$$\begin{array}{ccc} p_{n}\left(\psi \right) & = & \int_{0}^{T}g\left(\psi |\psi_{0}\right)p_{n-1}\left(\psi_{0}\right)\;d\psi_{0} \end{array}$$ (21)

$$\begin{array}{ccc} & ≔ & Lp_{n-1} \end{array}$$ (22)

The phase density *p*(*ψ*) is the fixed point of this map [48]:

$$\begin{array}{c} p(\psi )=Lp \end{array}$$ (23)

The ISI density is also given by:

$$\begin{array}{c} f\left(t\right)=\int_{0}^{T}f\left(t|\psi \right)p\left(\psi \right)\;d\psi \end{array}$$ (24)

The firing rate is

$$\begin{array}{c} \nu ={\left(\int_{0}^{\infty }tf\left(t\right)\;dt\right)}^{-1}. \end{array}$$

With the phase density *p*(*ψ*), we can calculate the vector strength (recall Eq (3)):

$$\begin{array}{c} r=\sqrt{{\left|\int_{0}^{T}p\left(\psi \right)\cos\left(2\pi \varphi \psi \right)\;d\psi \right|}^{2}+{\left|\int_{0}^{T}p\left(\psi \right)\sin\left(2\pi \varphi \psi \right)\;d\psi \right|}^{2}}. \end{array}$$ (25)

Although the expressions for the density of spike times *f*(*t*) and vector strength *r* are theoretically exact, it is quite difficult to compute numerically because the discretization of both *t* and *ω* necessarily have finite mesh sizes *dω* > 0, *dt* > 0, introducing numerical errors with each integral (transform). We thus altered the mesh sizes and domains for each noise value *D* and insured numerical accuracy of Eqs (24) and (25) by insuring the results matched the Monte Carlo simulation of the phenomenological system with the prescribed hazard functions (Eqs (6) and (16)).

### Monte carlo simulations

The results of the Monte Carlo simulations for the Fitzhugh-Nagumo model (Eqs (1) and (2)) were noisy. These simulations consisted of a network of 20,000 uncoupled cells run for a total 100 s of biological time. We used an Euler-Maruyama scheme [49] with a time step of 0.01 ms. The firing statistics (*ν* and *p*(*ψ*)) were calculated by averaging over time and over the uncoupled cells. For illustration purposes, we smoothed the simulated curves in Figs 1c and 2bi with the freely available function fastsmooth (see [50]) with a pseudo-Gaussian method because of the small time steps. Note that the curves in Fig 2bii and 2d were not smoothed (i.e., the raw values are plotted).

Computer code is available at http://doi.org/10.21974/V4159N.

## Supporting information

S1 Text Supplemental figures for “noise-enhanced coding in phasic neuron spike trains”. Fig A, Comparing various STAs of Fitzhugh-Nagumo model with different noise levels. Fig B, The STAs of Fitzhugh-Nagumo model with sinusoidal input. Fig C, Raw STA of Fitzhugh-Nagumo with no sinusoidal input.
(PDF)
