# Supplementary material for: Noise-enhanced coding in phasic neuron spike trains
Source: PLoS One. 2017 May 4;12(5):e0176963. doi: 10.1371/journal.pone.0176963 (PMC5417623; doi:10.1371/journal.pone.0176963)
Supplement: S1 Text — Fig A, Comparing various STAs of Fitzhugh-Nagumo model with different noise levels. Fig B, The STAs of Fitzhugh-Nagumo model with sinusoidal input. Fig C, Raw STA of Fitzhugh-Nagumo with no sinusoidal input. (PDF) [file pone.0176963.s001.pdf]

# Supplemental Figures for "Noise-Enhanced Coding in Phasic Neuron Spike Trains"

Cheng Ly[†,*] ; Brent Doiron[#,‡]

†*Department of Statistical Sciences and Operations Research, Virginia Commonwealth University, Richmond, VA 23284 U.S.A.*
*# Department of Mathematics, University of Pittsburgh, Pittsburgh, PA 15260 U.S.A.*
‡*Center for the Neural Basis of Cognition, Pittsburgh, PA 15213, U.S.A.*
∗ E-mail: CLy@vcu.edu

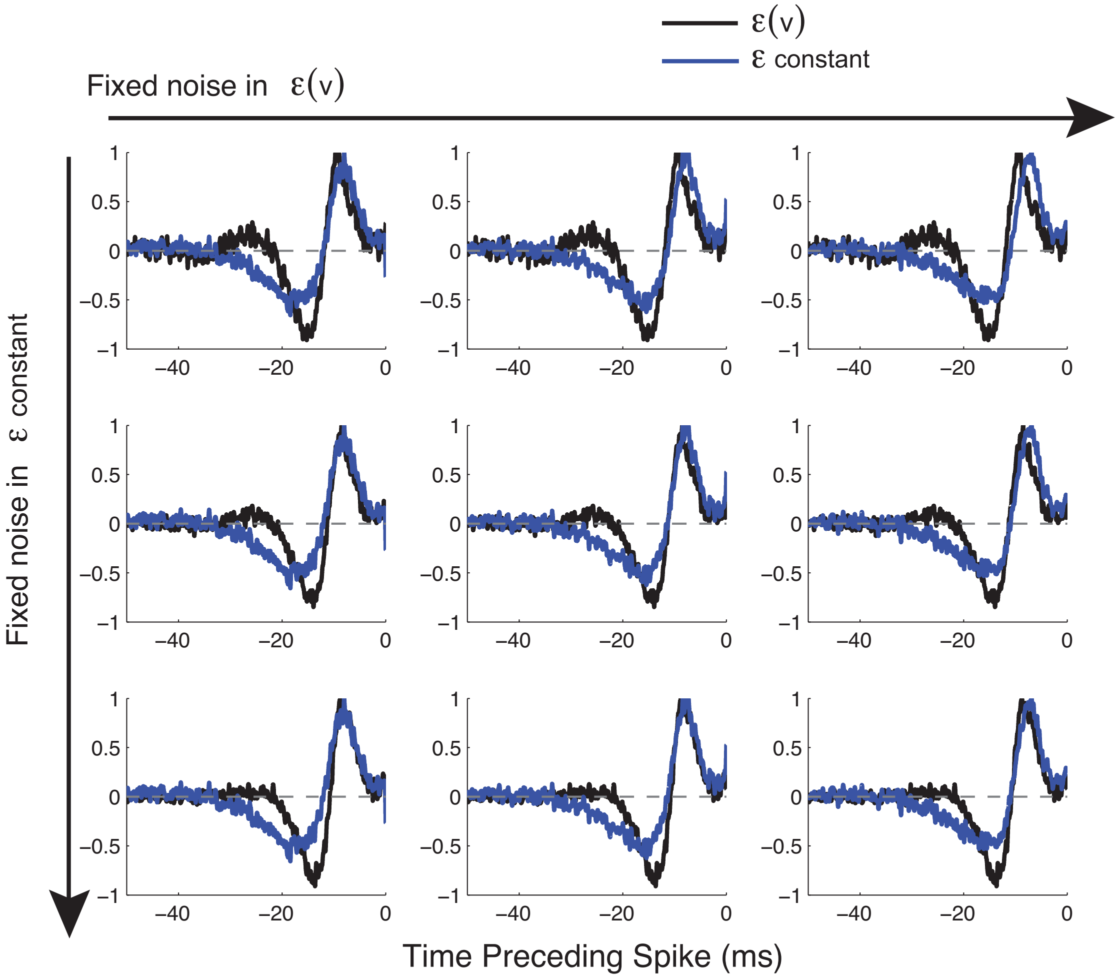


Figure A: **Comparing various STAs of Fitzhugh-Nagumo model with different noise levels.** Three different noise values were considered for the $\varepsilon(v)$ model (black) $D \in \{0.03, 0.04, 0.05\}$ (top, middle and bottom row, respectively) and for the $\varepsilon$ constant (blue) model $D \in \{0.049, 0.059, 0.069\}$ (left, center, and right column, respectively). In all cases, we see that the $\varepsilon(v)$ model (black) prefers sharper hyperpolarization, followed by depolarization; this is a key signature in the noise-enhanced coding of low frequency signals. STAs were normalized to have peak height of 1.

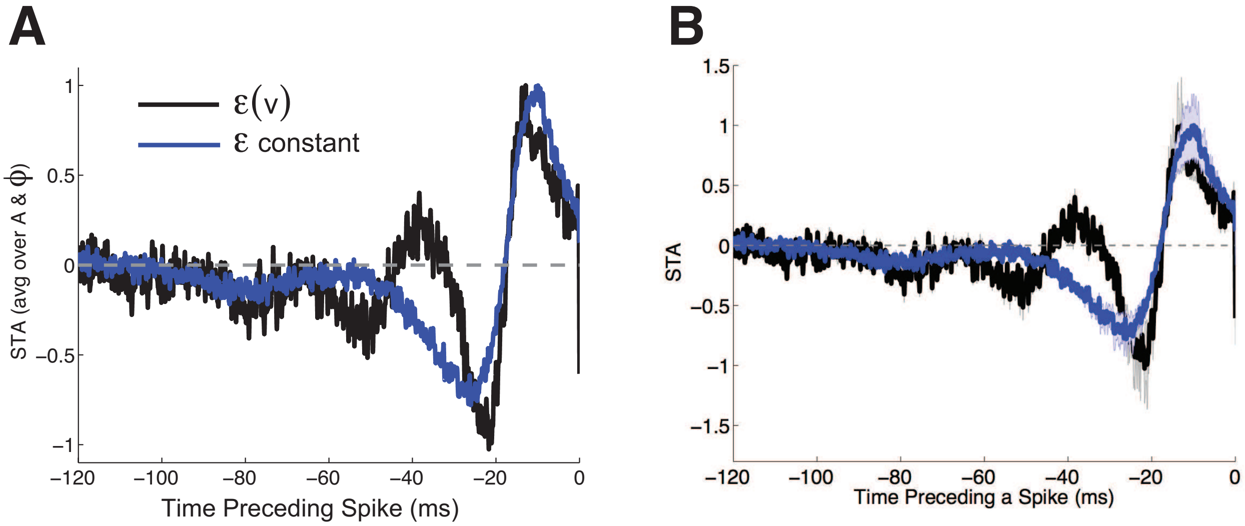


Figure B: **The STAs of Fitzhugh-Nagumo model with sinusoidal input.** Three different sinusoidal amplitudes $A \in \{0.01, 0.0.2, 0.03\}$ and frequencies $\varphi \in \{0.01, 1/150, 0.005\}$ were considered $I(t) = A\sin(2\pi\varphi t)$ with same noise levels as in Fig A. A) Showing the average STA over all combinations considered, and then normalizing so the height is 1. B) Same as A) but with shaded error bars (standard deviation over different parameter sets). The disparity between the $\varepsilon(v)$ model and $\varepsilon$ constant model is not as pronounced as when $I(t) = 0$, but is still apparent.

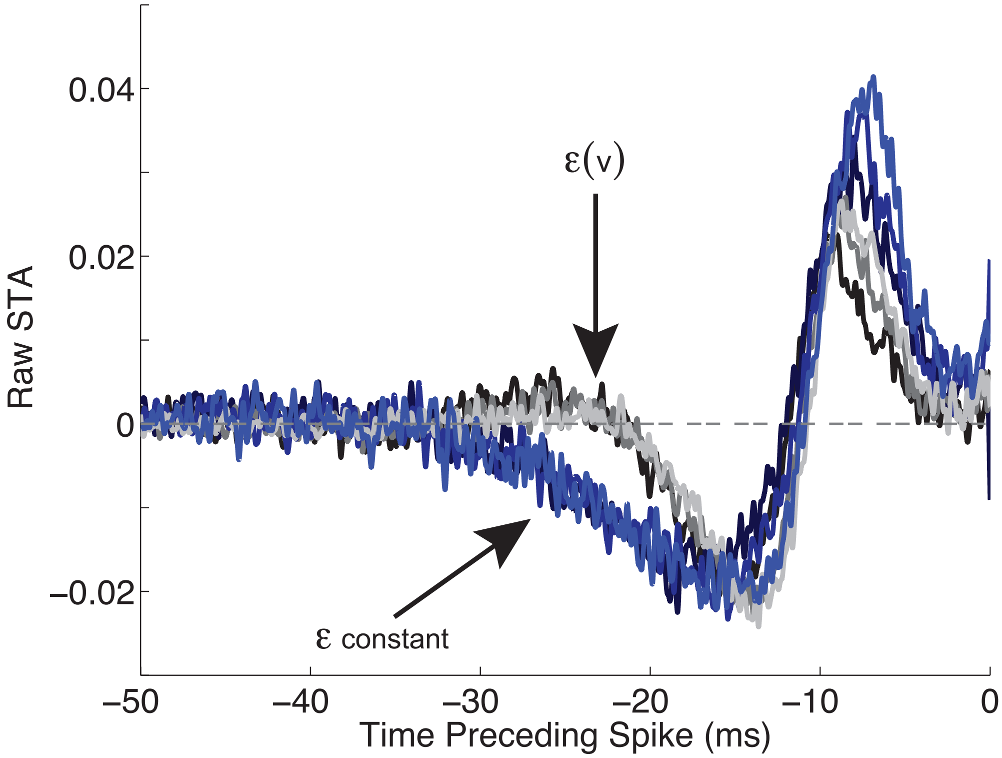


Figure C: **Raw STA of Fitzhugh-Nagumo with no sinusoidal input.** The unscaled STAs for the same noise levels in as in A Fig. As noise increases, there is not much difference until ≈15 ms before a spike; as the noise value increases, the peak of the STA decreases.
